# Supplementary material for: Combination of ultra-rapid DNA purification (PURE) and loop-mediated isothermal amplification (LAMP) for rapid detection of Trypanosoma cruzi DNA in dried blood spots
Source: PLoS Negl Trop Dis. 2023 Apr 14;17(4):e0011290. doi: 10.1371/journal.pntd.0011290 (PMC10132660; doi:10.1371/journal.pntd.0011290)
Supplement: S1 Table — (PDF) [file pntd.0011290.s001.pdf]

S1 Table: Step-by-step protocol

STEP	PROCEDURE
1	Define two bench zones for sample preparation and amplification area
2	Use two pair of gloves and clean carefully all surfaces with 1% sodium hypochlorite solution
3	Sodium hypochlorite must be in contact to surfaces at least 5 min
4	Remove excess sodium hypochlorite with water and dry using a paper towel
5	Transfer the sample* + 30 µL NaCl 334mM[§] into Heating Tube[§]
6	Mix by inverting 3 to 5 times
7	Remove the external pair of gloves
8	Incubate the heating tubes 5 min at 75 °C in a dry heater
9	Remove the cap ring from the top of the Adsorbent Tube[§] and screw the heating tube into the adsorbent tube
10	Mix vigorously, 10 times vertically and 10 times horizontally, until completed suspension of the absorbent powder
11	Cover the Injection Cap[§] with the cap ring and screw it into the bottom of the adsorbent tube
12	Squeeze the PURE device to drop the DNA eluate into the LAMP reaction tube[#] up to the middle of the two lines (approx. 30 µL)[¶]
13	Close immediately the cap sample-by-sample
14	Add 30 µL Positive Control** to LAMP reaction tube
15	Turn the tubes upside down and place the reaction tubes cap-side down on the bench for 2 min to dissolve the dried LAMP reagent inside the tube cap
16	Mix by inverting 5 times and then shake the reaction tubes downwards to collect the solution into the bottom of the tubes
17	Immediately place the reaction tubes into the incubator or heating-block during 40 min at 65 °C and 5 min at 80 °C
18	Read the reaction by naked eye or blue led visor light

* Sample: 30 µL heparin blood or 6-mm FTA or 30 µL Negative Control**

[§] PURE system: the kit provides 90 Heating Tubes, 90 Adsorbent Tubes, 90 Injection Caps, 2 tubes of 334 mM NaCl solution.

[#] LAMP kit: the kit has 48 reaction tubes (6 strips x 8 tubes) with dried LAMP reagent inside the tube cap, 3 tubes of Negative Control, one tube of Positive Control.

[¶] Right-handed operators should perform this step from right to left along the LAMP strip, whereas left-handed operators should perform it in the opposite direction, to avoid contaminating open LAMP microtubes.
