# Supplementary material for: Combination of ultra-rapid DNA purification (PURE) and loop-mediated isothermal amplification (LAMP) for rapid detection of Trypanosoma cruzi DNA in dried blood spots
Source: PLoS Negl Trop Dis. 2023 Apr 14;17(4):e0011290. doi: 10.1371/journal.pntd.0011290 (PMC10132660; doi:10.1371/journal.pntd.0011290)
Supplement: Attachment — Submitted filename: RESPONSES TO THE REVIEWERS.pdf [file pntd.0011290.s002.pdf]

RESPONSES TO THE REVIEWERS

Reviewer #1: The parasitemia in congenital Chagas disease could be less than 1 parasite equivalents/mL, the authors assessed LOD up to 5 and 10 parasite equivalents/mL, why?

Response: In this work we have focused on achieving an extraction method that can be used for point-of-care diagnosis and we have evaluated the sensitivity of LAMP using different type of samples. For this PURE-LAMP system we have tested up to 0.5 parasites/mL and the lowest concentration that rendered LAMP positive results was around 5-20 parasites/mL depending on the support used and on the parasite strain. The current diagnostic algorithm involves a parasitological test (microhematocrite or micromethod), which has a variable analytical sensitivity from 40 to 1000 parasites/ml, depending on the operators` skills, number of capillary tubes used and time of observation (Freilij et al, 1983; Bustos et al, 2019), reaching a clinical sensitivity of no more than 50% in most studies. Another important point of the method that we are evaluating is the sample volume, i.e., 30 µL for blood in heparin or a 6-mm FTA spot containing around 10 µL of blood (Hewawasam et al., 2018). In contrast, the reported LOD values below 1 parasite/mL (Besuschio et al, 2017), were based on 1 ml of peripheral or umbilical cord blood as starting material. Furthermore, at that time we used about 200 µL of sample for DNA extraction, using more laborious extraction methods (High Pure PCR template kits from Roche or Qiagen) than the ultra-rapid DNA extraction procedure described in the present work. To detect *T.cruzi* DNA in 10 to 30 µL of starting blood, we should expect that the patient harbors at least 1 parasite cell in the mentioned volume. After PURE extraction, we must load 30 µL of the eluate, which represents around 30% of the total DNA yield, so, theoretically, we can only detect samples harboring at least 10 parasites/mL, under the above-mentioned conditions. In most health centers of Argentina, the algorithm for diagnosis of congenital infection involves two timepoints for parasitological or molecular testing, one at birth and the other one at around 4-8 weeks of life. It has been observed for some infected babies that their parasitemia increases from time of birth to the first months of life, so the opportunity to detect infection increases too. Therefore, taking all this in account, although the LAMP sensitivity using the PURE procedure is lower than 1 par/mL in our hands, it is yet better than that of the parasitological methods and using lower volumes of blood, and accordingly, in our opinion it is worthwhile to be further evaluated in field studies.

Why was the inclusivity just determined with 20 and 50 parasite equivalents/mL?

Response: In order to evaluate the inclusivity of LAMP for three strains representative of circulating DTUs in humans, we chose two conditions for which we expected positive results, one concentration close to the LOD, which can be different in different strains, and another one for which we expect to have positive results for any strain.

Reviewer #2: In a previous study of the group, published in 2017, the LAMP kit prototype (by Eiken, Japan) for qualitative detection of *Trypanosoma cruzi* DNA was evaluated for analytical sensitivity and specificity using peripheral human blood as clinical sample, in comparison to a standardized qPCR. For this, LAMP sensitivity was estimated using DNA extracted with commercial kits or after “Boil & Spin” rapid preparation. Now, in the present study, authors are interested in the standardization and analytical evaluation of the performance of this LAMP test, as a point-of-care (POC) molecular test to be used for the

rapid diagnosis of congenital Chagas disease introducing FTA cards or Whatman 903 filter paper as solid supports for small-scale volumes of human blood. In addition, an ultrarapid purification system (PURE) for DNA extraction, also manufactured by Eiken, was evaluated by comparing artificially infected liquid blood in heparin as anticoagulant with different amounts of dried blood spot (DBS) either in FTA and Whatman 903 paper. A similar strategy using DBS coupled to PURE-LAMP as a point-of-care molecular test has been developed for malaria [Vincent et al. (2018) Combination of PURE-DNA extraction and LAMP-DNA amplification methods for accurate malaria diagnosis on dried blood spots. Malar J 17, 373].

The study design is appropriate to address the objectives.

There is no mention to Ethics statement regarding the use of seronegative human blood to obtain the spiked blood samples.

Response: Thanks to the reviewer for this observation. We just have added in Materials and Methods the information on the ethical aspects of blood samples from healthy donors.

Minor issues:

DNA extraction using PURE system.
Considering that the study has a context of technological development for the implementation of a point-of-care diagnostic test and so that the standardized method can be replicated accurately, I suggest the authors to describe more clearly the step-by-step extraction by the PURE system, even if it is included as supplementary material.

Response: Following this comment, despite PURE is a commercial DNA extraction kit, its use to detect *T.cruzi* DNA in fluid and FTA cards is an innovation. Accordingly, we have added a Table in the revised version of the manuscript (new S1 Table) containing a step-by-step description of the procedure for more clarity.

Raised questions:
Line 139. “mixed by shaking” – how many times, for how long (minutes)?

Response: Thanks to the reviewer for allowing us to clarify the procedure. In fact, the heating tube was mixed by inverting 3 to 5 times, following the manufacturer’s recommendation. This was added to the manuscript.

Lines 141-142. What is the approximate volume and concentration of the eluted DNA? Was the DNA measured before use?

Response: The total volume of each PURE column is around 100 microliters, but only 30 microliters are eluted within each LAMP tube. Previous 260/280 measurements showed that DNA obtained from heparinized blood gave a mean concentration of 12.9 ± 2.0 ng/ µL, Whatman 903 based DNA extractions gave 23.6 ± 2.6 ng/µL, while FTA cards rendered 30.5 ± 3.0 ng/ µL, in all cases calculated as single stranded DNA, because it is an alkaline extraction method. Although the concentration obtained using the three supports was different, the amount of DNA template in 30 microliters was adequate for amplification.

Moreover, FTA yielded low purity with protein contamination, but the LAMP technique was robust enough to amplify even with low quality DNA.

Lines 143-145. What is the reason to add a minor volume of NaCl in higher volumes of blood compared to DNA extraction from 30 uL of blood? It should be related to the maximal capacity of the heating tube, 60 uL? The calculations to arrive at the final concentration of 10mM NaCl is not clear, considering the mixture of 30 ul of 334 mM NaCl + 30 ul blood.

Response: Currently, PURE system is used for other infectious diseases, such as Tuberculosis and Malaria. In both types of samples (sputum or blood) the maximum volume added for DNA extraction is 60 µL. In the case of malaria, which sample is blood, the manufacturer's instructions recommend adding NaCl at a 10 mM final concentration, taking into account that the heating tube contains 1000 ml of volume. In order to increase the sensitivity of *T. cruzi* DNA detection, we tested more than 30 µL of blood plus NaCl until we reached the total volume of 60 µL, keeping the 10 mM NaCl final concentration in the reaction tube. This was to maintain the same conditions of the reaction proposed by the manufacturer.

Inclusivity and exclusivity.
Lines 172-174. Please, include “respectively” at the end of the sentence.

Response: Done

Analytical sensitivity and specificity.
Lines 184-186. Reference should be cited here [12 – Duffy et al., 2009] and also at the end of line 230 (Results).

Response: Done

Results</br></br>
-Does the analysis presented match the analysis plan?</br>
-Are the results clearly and completely presented?</br>
-Are the figures (Tables, Images) of sufficient quality for clarity?</br></br>

Reviewer #1: Line 205: The different experimental conditions should be explained in Methods section. Could be interesting to insert a paragraph about Study design.

Response: Following the recommendation of the reviewer, we have included a paragraph about Study design in the Methods section. Moreover, a Figure was added for clarity.

Line 211: What were the results using 40 and 50 µL of blood?
Response: We added this phrase in the new version of manuscript: Nevertheless*, the increase in sample volume did not result in a better sensitivity. Contrary to what was expected, an absence of amplification was obtained even at high concentrations of parasites*

per mL, indicating that the capacity limit of this system to remove contaminants and inhibitors from the blood is up to 30 µL of sample*. Thus*, the best results were obtained using 30 µL of blood plus 30 µL of 334 mM NaCl, so this starting volume was chosen for the following experiments.

Line 224: Figure 2 is not related to this part of the results.

Response: The mention to Figure 2 (Figure 3 in the revised version) and also Table 1 have been deleted.

line 226: In Methods, the authors indicate that inclusivity was evaluated using DNA from the 6 genotypes of T. cruzi, but they do not describe the results.
What were the results with T. rangeli?

Response: The paragraph about inclusivity and exclusivity was modified for clarity. In fact, in this work we only tested blood spiked with three strains belonging to different DTUs as well as a sample spiked with *Leishmania mexicana*. Other strains and *T.rangeli* had been previously tested by LAMP, using DNA purified by the High Pure PCR template preparation kits of Roche, as reported in Besuschio et al., 2017 [5].

Line 289: S1Table could be Table 2.

Response: Following your comment this Table has been incorporated to the main body of the manuscript. Because at the time of this review we had samples stored for up to one year and a half at room temperature, we decided to add stability results for a longer time than in the first version of the manuscript.

Line 310: What are the possible explanations for the false positives?

Using the PURE extraction method, Whatman 903 samples gave rise in some replicates to false positive findings, detected by fluorescence in the LAMP tubes containing DBS loaded with blood without parasites (negative sample controls), while FTA cards gave always specific non-detectable findings in the same non-spiked blood. Accordingly, putative laboratory contamination of the non-spiked blood with parasite DNA was discarded. This has been clarified in the revised Discussion. In general, Whatman 903 is a protein saved card. However, as it is used in the National Prenatal Screening program, we attempted to test its feasibility for LAMP. Unfortunately, the occurrence of some false positive results determined selecting FTA for further work.

Line 322: “which remains to be tested in our case”, why?

This sentence was modified, for clarity.

Reviewer #2: The results and figures are clearly presented.

Line 208. To correct the volumes of heparinized blood used, as mentioned in methods - DNA extraction using PURE system. Tested volumes were 30 to 50 µL.

Response: Apologizes for the mistake. We corrected the wrong value.

Line 218. What was the reason to add four times higher the volume of blood to the FTA cards compared to the 30 µL of dried blood included in each Whatman 903 spot?

Response: The circles of FTA cards have a diameter of 25 mm with a capacity of up to 125 µL of blood sample, whereas the circles of Whatman 903 have a diameter of 13 mm with a capacity of up to 50 µL of blood sample. In fact, we did not use four times higher volumes of blood in FTA than in Whatman. We took one 6 mm FTA disc with an approximate capacity of 10 µL of blood sample, according to Hewawasam et al [reference 20] and the whole bloodstain from Whatman has around 30 µL of blood. Considering the limitations of the PURE system in terms of the amount of sample that can be added, we seeded up to 30 µL of blood from Whatman discs and analyzed one or two DBSs per reaction. We obtained better sensitivity with two DBS but worse specificity (false positives), as it has been mentioned above.

Table 1: The difference in the volume of blood between both DBS supports did not interfere in the positivity results?
Response: As mentioned above, the difference in volume between both DBS supports was not such: it was around 10 µL vs 30 µL for FTA and Whatman, respectively. This was mentioned in the discussion of the manuscript. However, it cannot be discarded that this small difference in blood volumes has an effect in sensitivity.

Line 288. To include … obtaining in total four “positive” out of six LAMP results…
Response: Done

Conclusions</br></br>
-Are the conclusions supported by the data presented?</br>
-Are the limitations of analysis clearly described?</br>
-Do the authors discuss how these data can be helpful to advance our understanding of the topic under study?</br>
-Is public health relevance addressed?</br></br>

Reviewer #1: The conclusion can be improved.
The limitations are not described.
Inclusion the real clinical samples could improve the relevance of this study.

Response: We have improved the paragraph presenting the conclusions of this work. We have not included clinical samples, given that the results in clinical samples will be presented elsewhere, once the results from a prospective field study currently started will be available.

Reviewer #2: The conclusions are very well supported and no limitations of analysis were described. Procedures were standardized for the LAMP reactions from small volumes of liquid blood or the use of FTA sampling format for the detection of T. cruzi DNA. The use of Whatman 903 filter paper was discarded due to the inespecificity of results (false positive). To validate the PURE-LAMP method in the field, in endemic and non-endemic regions, operational studies with neonates born to seropositive women are planned as well as the use of this point-of-care test for rapid screening of infected cases in oral Chagas disease

outbreaks.

DISCUSSION.

Lines 320-322. A reference should be included.

Response: The reference has been added.

Lines 362-364. The phrase is related to the work by Hewawasam et al. (2018). However, in the present study each DBS was blotted with 125 µL blood in Whatman FTA cards, 4 times higher than the tested volume for liquid heparinized blood. Could the authors comment please?

Response: As we have explained above, we did not use the whole FTA card for PURE-LAMP testing; but we only used one 6-mm punched disc, which would contain around 10 µL of blood.

Editorial and Data Presentation Modifications?</br>
Use this section for editorial suggestions as well as relatively minor modifications of existing data that would enhance clarity. If the only modifications needed are minor and/or editorial, you may wish to recommend “Minor Revision” or “Accept”.

Reviewer #1: (No Response)

Reviewer #2: This reviewer has no editorial suggestion. The revision includes minor modifications, as follows:

METHODS: DNA extraction using PURE system.
Considering that the study has a context of technological development for the implementation of a point-of-care diagnostic test and so that the standardized method can be replicated accurately, I suggest the authors to describe more clearly the step-by-step extraction by the PURE system, even if it is included as supplementary material.

Line 139. “mixed by shaking” – how many times, for how long (minutes)?
Lines 141-142. What is the approximate volume and concentration of the eluted DNA? Was the DNA measured before use?
Lines 143-145. What is the reason to add a minor volume of NaCl in higher volumes of blood, compared to DNA extraction from 30 uL of blood? It should be related to the maximal capacity of the heating tube, of 60 uL? The calculation to arrive at the final concentration of 10mM NaCl is not clear, considering the mixture of 30 ul of 334 mM NaCl + 30 ul blood.
Lines 172-174. Please, include “respectively” at the end of the sentence.
Lines 184-186. Reference should be cited here [12 – Duffy et al., 2009] and at the end of line 230 (Results).

There is no mention to Ethics statement regarding the use of seronegative human blood samples to obtain the spiked blood.

Line 208. To correct the volumes of heparinized blood used, as mentioned in methods - DNA extraction using PURE system. Tested volumes were 30 to 50 µL.

Line 218. What was the reason to add four times higher the volume of blood to the FTA cards compared to the 30 µL of dried blood included in each Whatman 903 spot?
Table 1: The difference in the volume of blood between both DBS supports did not interfere in the positivity results between them?
Line 288. To include … obtaining in total four "positive" out of six LAMP results….

Lines 320-322. A reference should be included.
Lines 362-364. The phrase is related to the work by Hewawasam et al. (2018). However, in the present study each DBS was blotted with 125 µL blood in Whatman FTA cards, 4 times higher than the tested volume for liquid heparinized blood. Could the authors comment?

Response: All these questions have been already answered in the previous pages.

Summary and General Comments</br></br>
Use this section to provide overall comments, discuss strengths/weaknesses of the study, novelty, significance, general execution and scholarship. You may also include additional comments for the author, including concerns about dual publication, research ethics, or publication ethics. If requesting major revision, please articulate the new experiments that are needed.

Reviewer #1: The study describes the use of blood preservation on filter paper for its application in LAMP. But real clinical samples were not included. Since the limit of detection (LOD) of this methodology was 20 parasites/mL, lower parasitemias would not be detected. It would be convenient to point this out.

Response: This issue has been discussed in the revised version of the manuscript

Reviewer #2: The group is strong and experienced in the development of molecular tests for the detection of T. cruzi and lately has been working on improving the LAMP methodology as a point-of-care diagnostic tool for the early diagnosis of acute infections such as congenital Chagas disease. Overall the study is very well designed with clear presentation of the results and it is original from the point of view of improving the LAMP test for application in the point-of-care diagnostic of acute T. cruzi infections. In this sense, the use of FTA cards as solid support for small-scale volumes of human blood is imperative for the preservation and transport of clinical samples from patients with acute Chagas disease. Another novelty of the study refers to the standardization of the PURE system for DNA extraction developed by Eiken, Japan, which reasonably decreases the time for DNA obtention compared to the use of specific columns provided in commercial kits and which require several steps until the DNA elution.

Response: Thank the reviewer for these comments

It has to be highlighted the need to include Ethics statement for the use of human blood to obtain the spiked blood samples.

Response: An Ethics statement has been added in the Methods section.

PLOS authors have the option to publish the peer review history of their article (what does this mean?). If published, this will include your full peer review and any attached files.

If you choose “no”, your identity will remain anonymous but your review may still be made public.

Do you want your identity to be public for this peer review? For information about this choice, including consent withdrawal, please see our Privacy Policy.

Reviewer #1: No

Reviewer #2: Yes: Constança Britto

Figure Files:
While revising your submission, please upload your figure files to the Preflight Analysis and Conversion Engine (PACE) digital diagnostic tool, https://pacev2.apexcovantage.com. PACE helps ensure that figures meet PLOS requirements. To use PACE, you must first register as a user. Then, login and navigate to the UPLOAD tab, where you will find detailed instructions on how to use the tool. If you encounter any issues or have any questions when using PACE, please email us at figures@plos.org.

Data Requirements:
Please note that, as a condition of publication, PLOS' data policy requires that you make available all data used to draw the conclusions outlined in your manuscript. Data must be deposited in an appropriate repository, included within the body of the manuscript, or uploaded as supporting information. This includes all numerical values that were used to generate graphs, histograms etc.. For an example see here: http://www.plosbiology.org/article/info%3Adoi%2F10.1371%2Fjournal.pbio.1001908#s5.

Reproducibility:
To enhance the reproducibility of your results, we recommend that you deposit your laboratory protocols in protocols.io, where a protocol can be assigned its own identifier (DOI) such that it can be cited independently in the future. Additionally, PLOS ONE offers an option to publish peer-reviewed clinical study protocols. Read more information on sharing protocols at https://plos.org/protocols?utm_medium=editorial-email&utm_source=authorletters&utm_campaign=protocols

References
Please review your reference list to ensure that it is complete and correct. If you have cited papers that have been retracted, please include the rationale for doing so in the manuscript text, or remove these references and replace them with relevant current references. Any

changes to the reference list should be mentioned in the rebuttal letter that accompanies your revised manuscript. If you need to cite a retracted article, indicate the article's retracted status in the References list and also include a citation and full reference for the retraction notice.
